# Supplementary material for: Comparative multiomics analysis of cell physiological state after culture in a basket bioreactor
Source: Sci Rep. 2022 Nov 23;12:20161. doi: 10.1038/s41598-022-24687-4 (PMC9686226; doi:10.1038/s41598-022-24687-4)

99109268:-@NW_023666037.1:199105168:199105295:-@NW_023666037.1:199098931:199099191:-@NW_02366

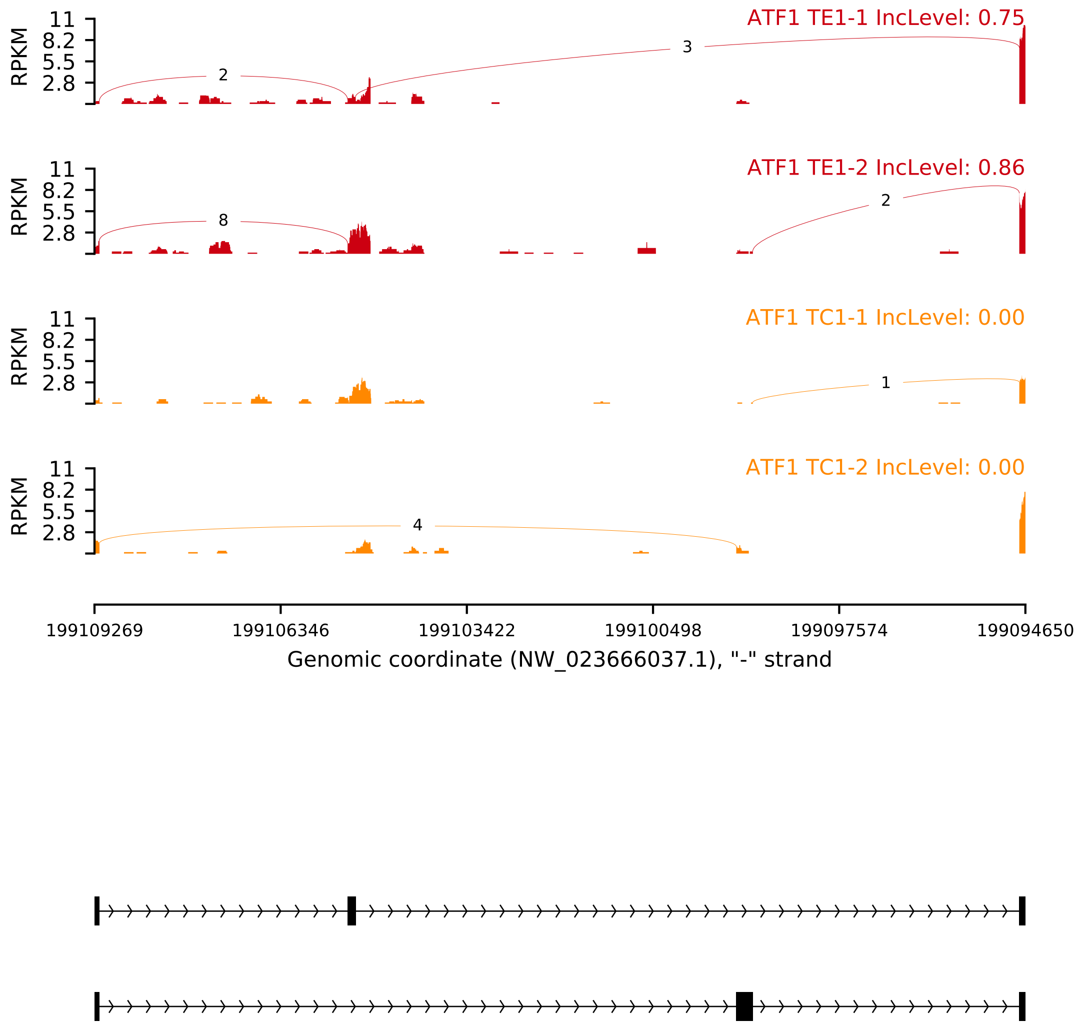

Supplement: Supplementary file 1 — Supplementary Information 1. [file 41598_2022_24687_MOESM1_ESM.zip › raw data/Transcriptomic raw data/7.AS/2.dasplot/TE1vsTC1/MXE/2_ATF1_NW_023666037.1_199109192_199109268_-@NW_023666037.1_199105168_199105295_-@NW_023666037.1_199098931_199099191_-@NW_023666037.1_199094648_199094746_-.pdf]
